# Berberine: A natural modulator of immune cells in multiple sclerosis

**DOI:** 10.1002/iid3.1213

**Published:** 2024-03-13

**Authors:** Esmaeil Yazdanpanah, Sepehr Dadfar, Alireza Shadab, Niloufar Orooji, MohammadHossein Nemati, Alireza Pazoki, Seyed‐Alireza Esmaeili, Rasoul Baharlou, Dariush Haghmorad

**Affiliations:** ^1^ Immunology Research Center Mashhad University of Medical Sciences Mashhad Iran; ^2^ Department of Immunology, School of Medicine Semnan University of Medical Sciences Semnan Iran; ^3^ Cancer Research Center Semnan University of Medical Sciences Semnan Iran

**Keywords:** berberine, dendritic cells, multiple sclerosis, Th1 and Th17 cells, Th2 and Treg cells

## Abstract

Berberine is a benzylisoquinoline alkaloid found in such plants as *Berberis vulgaris, Berberis aristata*, and others, revealing a variety of pharmacological properties as a result of interacting with different cellular and molecular targets. Recent studies have shown the immunomodulatory effects of Berberine which result from its impacts on immune cells and immune response mediators such as diverse T lymphocyte subsets, dendritic cells (DCs), and different inflammatory cytokines. Multiple sclerosis (MS) is a chronic disabling and neurodegenerative disease of the central nervous system (CNS) characterized by the recruitment of autoreactive T cells into the CNS causing demyelination, axonal damage, and oligodendrocyte loss. There have been considerable changes discovered in MS regards to the function and frequency of T cell subsets such as Th1 cells, Th17 cells, Th2 cells, Treg cells, and DCs. In the current research, we reviewed the outcomes of in vitro, experimental, and clinical investigations concerning the modulatory effects that Berberine provides on the function and numbers of T cell subsets and DCs, as well as important cytokines that are involved in MS.

## Graphical abstract

There have been considerable changes discovered in multiple sclerosis (MS) regards to the function and frequency of T cell subsets such as Th1 cells, Th17 cells, Th2 cells, Treg cells, and dendritic cells (DCs). In the current research, we reviewed the outcomes of in vitro, experimental, and clinical investigations concerning the modulatory effects that Berberine provides on the function and numbers of T cell subsets and DCs, as well as important cytokines that are involved in MS.

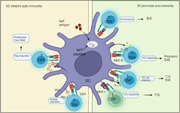

## 1 INTRODUCTION

Multiple sclerosis (MS) is an autoimmune and neurodegenerative disease that affects about 2.5 million people in the world; a chronic neuroinflammatory disease that causes a disability in young people with an average age of 30 years, and women are at much higher risk of MS than men^1^; the prevalence of this disease is 2 in 100,000 in Japan, 100 in 100,000 in Northern Europe^2^ and 29 in 100,000 in Iran.^3^ MS is one of the leading causes of disability in the United States.^4^

Despite the mechanism of affecting MS remains unknown, some genetics and environmental factors can increase susceptibility to this disease; for instance, being heterozygote for genetic locus HLA‐DRB1*15:01 has a chance proportion of MS above 3 and homozygote of it increases this ratio to above 6^5^; environmental factors including vitamin D deficiency, being a daily smoker and EBV infection. Symptoms of MS vary from central paralysis to difficulty walking, visual problems, fatigue, hearing loss, Amblyopia (lazy eyes), and even sexual impotence.^3^, ^6^ The economy is negatively impacted by this disease because the ability to work in patients goes from 82% to 8%.^7^

The exact pathology of MS has not yet been discovered but based on multiple investigations, the main cause of MS is the infiltration of immune cells like T cells, B cells, and macrophage cells in the central nervous system (CNS) and starting inflammatory and immune reactions that lead to demyelination and axon loss in the white matter of the brain and spinal cord that disrupts the neuroaxonal signals^1^, ^5^, ^8^; the main treatment for MS has not been discovered yet, but the base of the existing agents are: anti‐inflammatory, immunomodulatory and immunosuppression drugs.^1^, ^8^

The immunopathology of MS can be explained by the study on mice in which MS is induced. In the experimental autoimmune encephalomyelitis (EAE), an animal model of MS, healthy mice are converted into MS mice in two different ways; first: injection of CNS antigens with immune activators,^1^ second: adoptive transfer of activated T cells from EAE mice to healthy mice^9^; study on them has shown that various immune cells, such as T cells, B cells, macrophages, microglia, dendritic cells (DCs), monocytes, NKT cells, and other cells play an important role in the pathogenesis of the disease.^10^, ^11^

**Figure 1:**
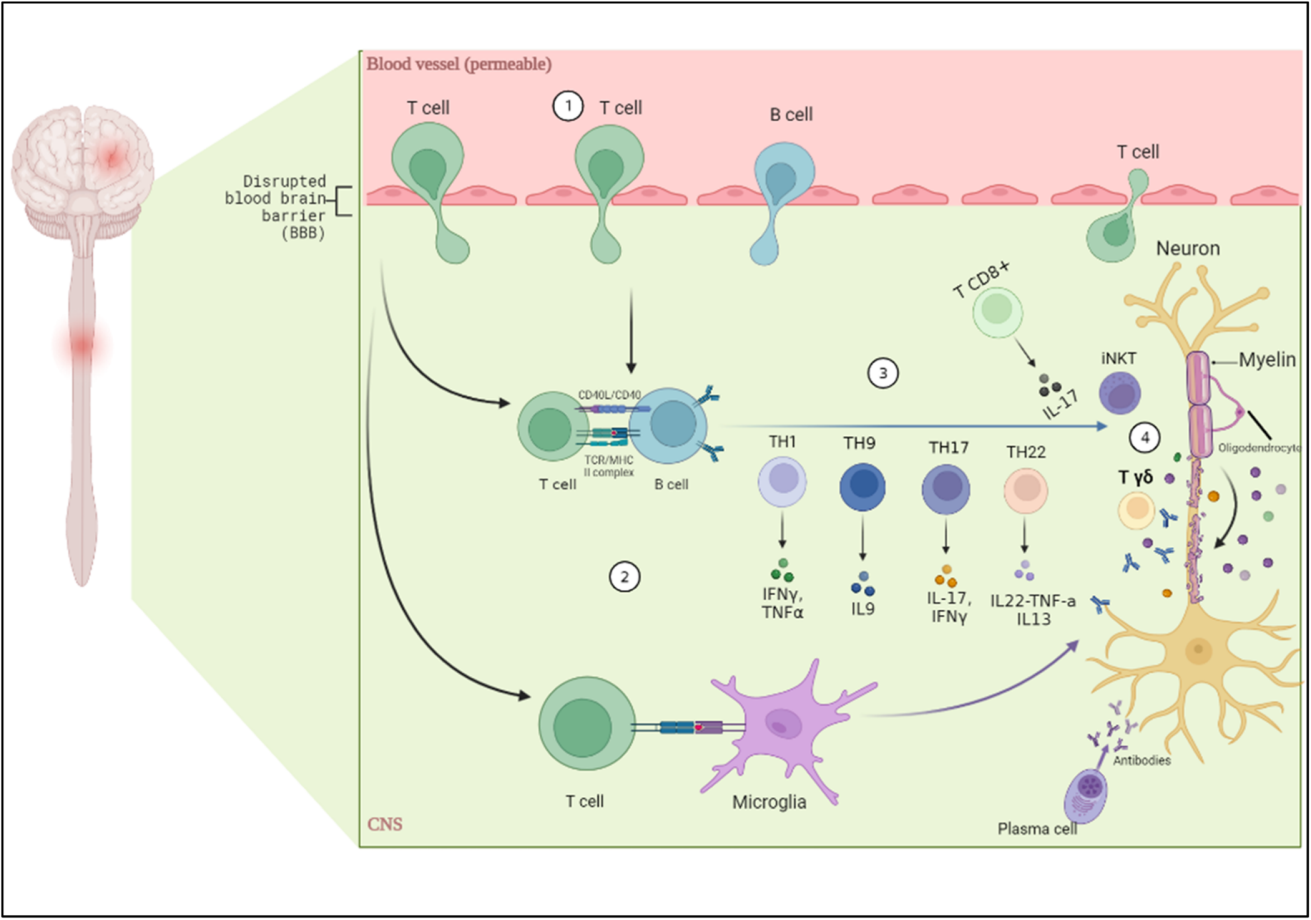
Immunopathology of multiple sclerosis: (1) blood–brain barrier (BBB) became permeable to lymphocytes. (2) T cell interacted with B cells and microglia to activate them. (3) Antibodies and cytokines released; antibodies released from plasma cells and cytokines were released from T cells. Th1 mainly released IFN‐γ and TNF‐α, Th9 released IL‐9, Th17 released IL‐17 and IFN‐γ, Th22 released IL‐22, TNF‐α and IL‐13 and T CD8^+^ released IL‐17. (4) With these cytokines and cells demyelination of neurons occurred.

The main cells that lead to MS pathogenesis are adaptive immune cells; T CD4^+^ like Th1 and Th17 that became auto‐reactive in the peripheral sites like lymph nodes, leading to demyelination by infiltrating the CNS and also secreting their specific pro‐inflammatory cytokines^11^, ^12^; T CD8^+^ have a much higher number at the lesion sites because MHC class I molecules, which present antigens to these cells, is expressed in all nucleated cells; since Treg cells have a role in tolerance, when they are disrupted, immune cells become reactive^10^; B cells have a varied role in the progression of MS; they can activate T cells by processing and presenting antigens and also producing antibodies by plasma cells,^5^, ^11^ which one way of treating MS is anti‐CD20 treatment strategies (rituximab) that cause the depletion of B cells and a small population of circulating Th1 and Th17^13^ (Figure 1).

Innate immune cells play a significant role in the pathogenesis of MS; for instance: monocytes and macrophages secrete pro‐inflammatory cytokines, DCs present antigens to T cells and activate them, whereas NKT cells shift the immunological balance toward Th2 cells that can help in healing the disease.^10^, ^11^

## 2 BERBERINE AND ITS BIOLOGICAL EFFECTS

In recent years, tremendous attention has been given to natural products due to their specific pharmacological activities for clinical applications in different national healthcare settings.^14^ Of these various compounds, Berberine with a molecular formula of C20H18NO4 and a molar mass of 336.36 g/mol has emerged as a compound with extensive biological properties.^14^ Berberine is an isoquinoline alkaloid derivative found in the roots, rhizomes, and stem bark of plants within a variety of genera, including Annonaceae, Berberidaceae, Menispermaceae, Papaveraceae, Ranunculaceae, Rutaceae, and many others.^15^ Interestingly, Berberine appeared to be almost safe in usual doses, with a relatively low incidence of side effects.^16^, ^17^ Berberine is a yellow odorless crystalline powder with an extremely bitter taste that is slightly soluble in ethanol or methanol, and sparingly soluble in water.^16^

Berberine, a traditional Chinese medicine, has long been used as an effective drug in treating metabolic disorders, and gastrointestinal, and endocrine diseases such as obesity, hypertension, fatty liver, type‐2 diabetes, hyperlipidemia, diarrhea, and gout in humans and animals.^16^, ^18^ Besides, accumulating evidence during the last two decades has addressed the polytrophic pharmacological potential of Berberine including antimicrobial, antioxidant, antitumor, anti‐cardiovascular, antidiabetic properties, anti‐hypertensive, immunosuppressive, anti‐inflammatory, and neuroprotective activities.^19^, ^20^

Notably, the potential of Berberine in controlling different autoimmune disorders has been assessed in several experimental studies and clinical trials.^21^ As an anti‐inflammatory and immunosuppressive, Berberine efficacy inhibits the inflammatory responses involved in clinically apparent autoimmune diseases MS, rheumatoid arthritis, psoriasis, and inflammatory bowel disease in human or animal models.^21^

**Figure 2:**
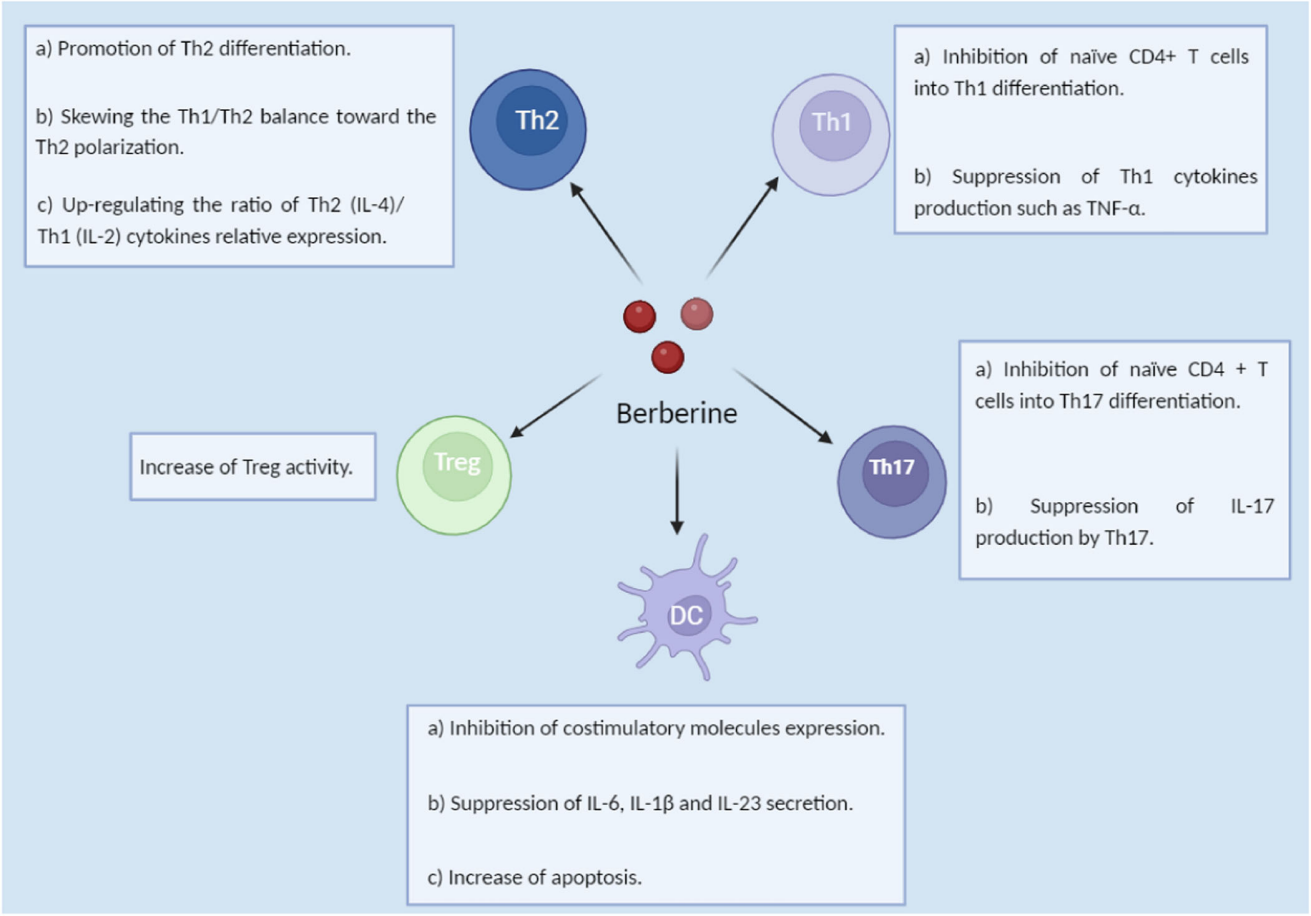
The overall effects of Berberine on immune cells: Berberine can regulate immune cells in different ways to moderate multiple sclerosis.

The related mechanisms of Berberine against autoimmune diseases and associated inflammatory disorders mainly involve inhibiting inflammation, modulation of Th17/Treg balance favor to Treg, and modulation of Th1/Th2 balance favor to Th2.^21^, ^22^, ^23^ Of note, this effect is accompanied by inhibiting pro‐inflammatory mediators and upregulating anti‐inflammatory mediators^20^, ^21^, ^22^ (Figure 2).

## 3 ROLE OF DCs IN THE PATHOGENESIS OF MS AND MUDULATORY EFFECTS OF BERBERINE ON DCs

Antigen‐presenting cells (APCs) process and present antigens on MHC molecules for recognition by T cells via the TCR. DCs as a special subclass of APCs are important for the initiation and regulation of adaptive immunity, as well as in the triggering of autoimmunity.^24^, ^25^ Human DCs have six subsets including blood versus tissue DCs, plasmacytoid DCs (pDCs), classical DCs (cDCs), CD1a^+^ CD14^+^ tissue DCs, Langerhans cells, and monocyte‐derived DCs.^26^

DCs have crucial roles in the development of the T cell repertoire as well as the activation and polarization of myelin‐specific T cells in the peripheral and CNS in the context of MS and its model EAE.^27^ MS is a complex autoimmune disease in which leukocytes, especially DCs infiltrate into the CNS of patients, causing demyelination and axonal damage as a result.^28^

Anatomic and flow cytometry studies relying on surface markers characteristic of peripheral DCs, have identified the presence of DCs in the CNS as indisputable.^29^, ^30^ There are differences in the increasing presence and activation of DCs in the CNS of MS patients. Studies showed that the average number of DCs within the CSF was increased but it was not related to the severity of the disease as well as more DCs were seen in patients in the early phases of optic neuritis. In addition, CD11b^+^ DCs in CSF became more activated with increasing levels of costimulatory molecules and MHC class II.^31^, ^32^

**Figure 3:**
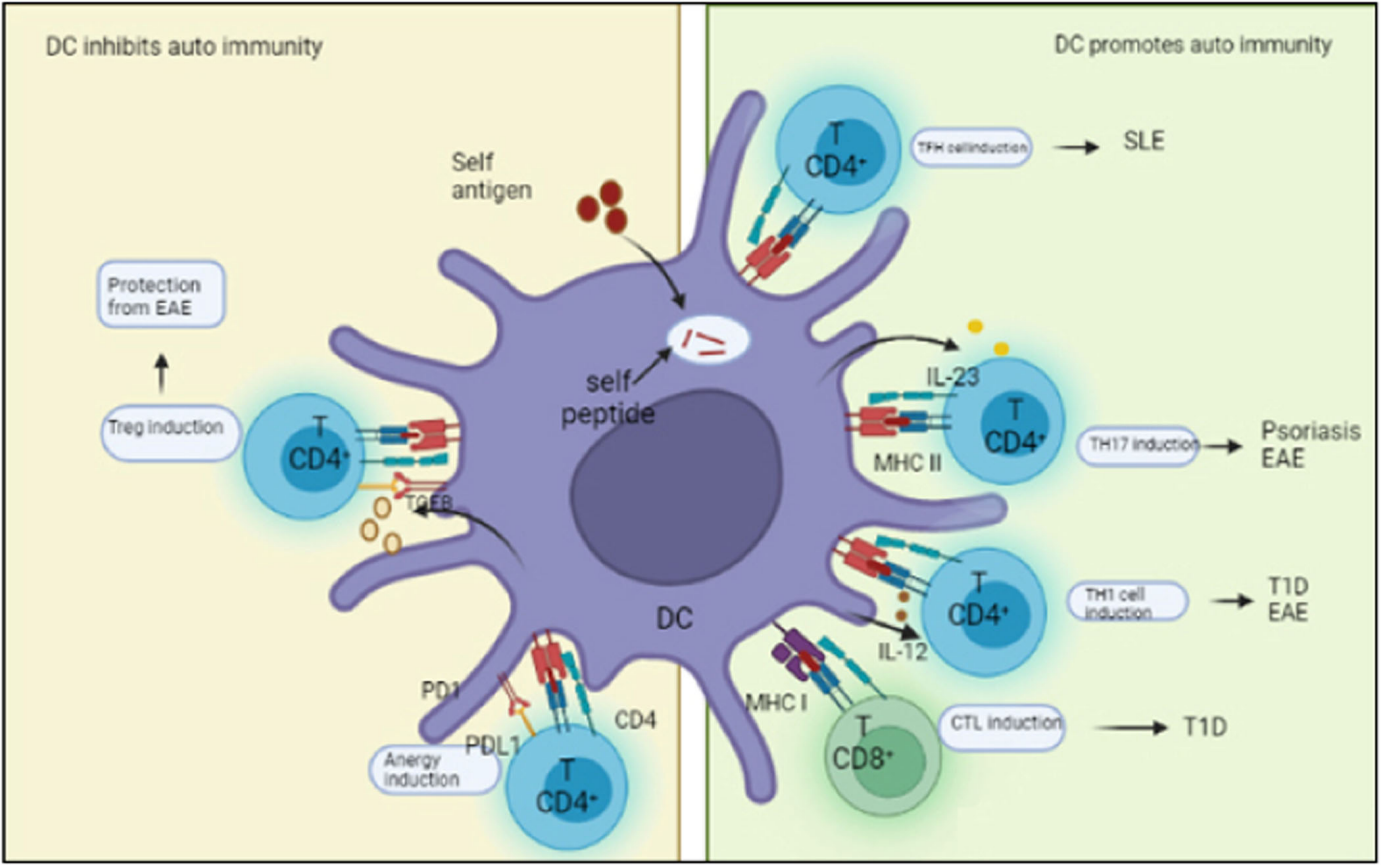
Roles of DCs in autoreactive T cell responses. Presentation of self‐antigens to T cells in the context of programmed cells, the interaction between PD1 and PDL1 and TGF‐β signaling cause Anergy of autoreactive T cells can promote their development into Treg. In contrast, presentation of self‐antigens to T cells in the context of pro‑inflammatory mediators such as IL‑6, IL‑12, and IL‑23 promotes the development of self‑reactive effector CD4^+^ T cells and CTLs. These self‑reactive T cells might cause pathological autoimmune diseases, including EAE in mice, or systemic lupus erythematosus (SLE), psoriasis, and Type 1 diabetes (T1D) in patients. DC, dendritic cell.

There are some abnormal characteristics of circulating DC precursors and DCs from the blood of MS patients such as producing more TNF‐α, IL‐6, and IFN‐γ. Moreover, the blood DCs of MS patients showed a pro‐inflammatory profile including more IL‐12 and TNF‐α production along with costimulatory molecule upregulation.^33^, ^34^ Furthermore, differences in DC function were found such as the capability of polarizing CD4^+^T cells to ward IFN‐ γ production in a mixed lymphocyte reaction and an elevation of IL‐23 secretion from monocyte‐derived DCs suggests supporting abnormal Th17 polarization of T cells.^35^, ^36^ Another study showed that a reduction of blood CD11b^+^DCs was found in secondary progressive MS (SPMS) compared to relapsing‐remitting MS (RRMS)^37^ patients (Figure 3).

Experimental evidence demonstrated the importance of DCs as an initiator of auto‐reactive T‐cell responses.^38^ The role of DCs in MS pathogenesis is complicated because DCs are heterogeneous with a range of functional phenotypes.^39^ Plasmacytoid DCs have two different subsets including type 1 (pDC1) with high levels of CD123, low levels of CD86 and TLR2 which are the source of IFN‐α and induce IL‐10 producing T cells, and type 2 (pDC2) with low levels of CD123, high levels of CD86 and TLR2 that, secret IL‐6 and TNF‐α and direct naïve T cells toward IL‐17 secreting Th17 cells. The pDC1/pDC2 ratio in MS patients is a noteworthy shift to pDC2.^40^ Moreover, some mechanisms, related to DCs, such as type 1 IFN production by pDCs, might lead to pathogenesis in autoimmune disease.^41^ Furthermore, increases in the expression of the CCR5 in cDCs of CSF have been observed.^32^, ^42^

Abnormalities of DCs contribute to the pathology and response to treatment of MS. Considering the role of DCs in the pathogenesis of MS; they can be used as a therapeutic target in MS. Actually, the current immunomodulatory therapies used for the treatment of MS affect DC function.^43^ For example, PDL1, a molecule causes the inhibition of DCs signaling to T cells.^44^ Currently, Glatiramer acetate is used as an immunomodulatory drug to treat MS, which is associated decreasing in TNF‐α and IL‐12p70 production by monocyte‐derived DCs and reduced CD40 expression and pro‐inflammatory activity in pDCs.^45^

Mature DCs were found after LPS stimulation more sensitive to berberine than immature DCs. So, the expression of CD80/CD86 and IL‐12 was low in the presence of berberine in response to LPS stimulation.^46^ Some effects of berberine on mature DCs stimulated with LPS were shown as inhibition of expression of costimulatory molecules including CD80, CD86, and CD40. Furthermore, the production of IL‐23 was induced; in contrast, the levels of IL‐6 and IL‐1β were decreased.^47^ Also, berberine can activate DC apoptosis.^48^ It is necessary to mention that more studies should be done to investigate the effect of berberine on DC function and related mechanisms.

## 4 ROLE OF TH1 CELLS IN THE PATHOGENESIS OF MS

A subset of TCD4^+^ called Th1 cells is produced as a result of IL‐12 and IFN‐γ signals acting on naïve T cells.^49^, ^50^ Th1 is often identified by the expression of surface markers including CXC chemokine receptor type 3 (CXCR3) and IL‐12 receptor (IL‐12R), as well as the production of a transcription factor called T‐bet that is induced by IL‐12 and IFN‐γ signaling.^51^, ^52^, ^53^ Th1 can produce variant cytokines such as IFN‐γ, IL‐2, TNF‐α, and GM‐CSF.^54^, ^55^ IFN‐γ, the main cytokine generated by Th1 cells, causes macrophages to become activated and fight intracellular pathogens.^49^

**Figure 4:**
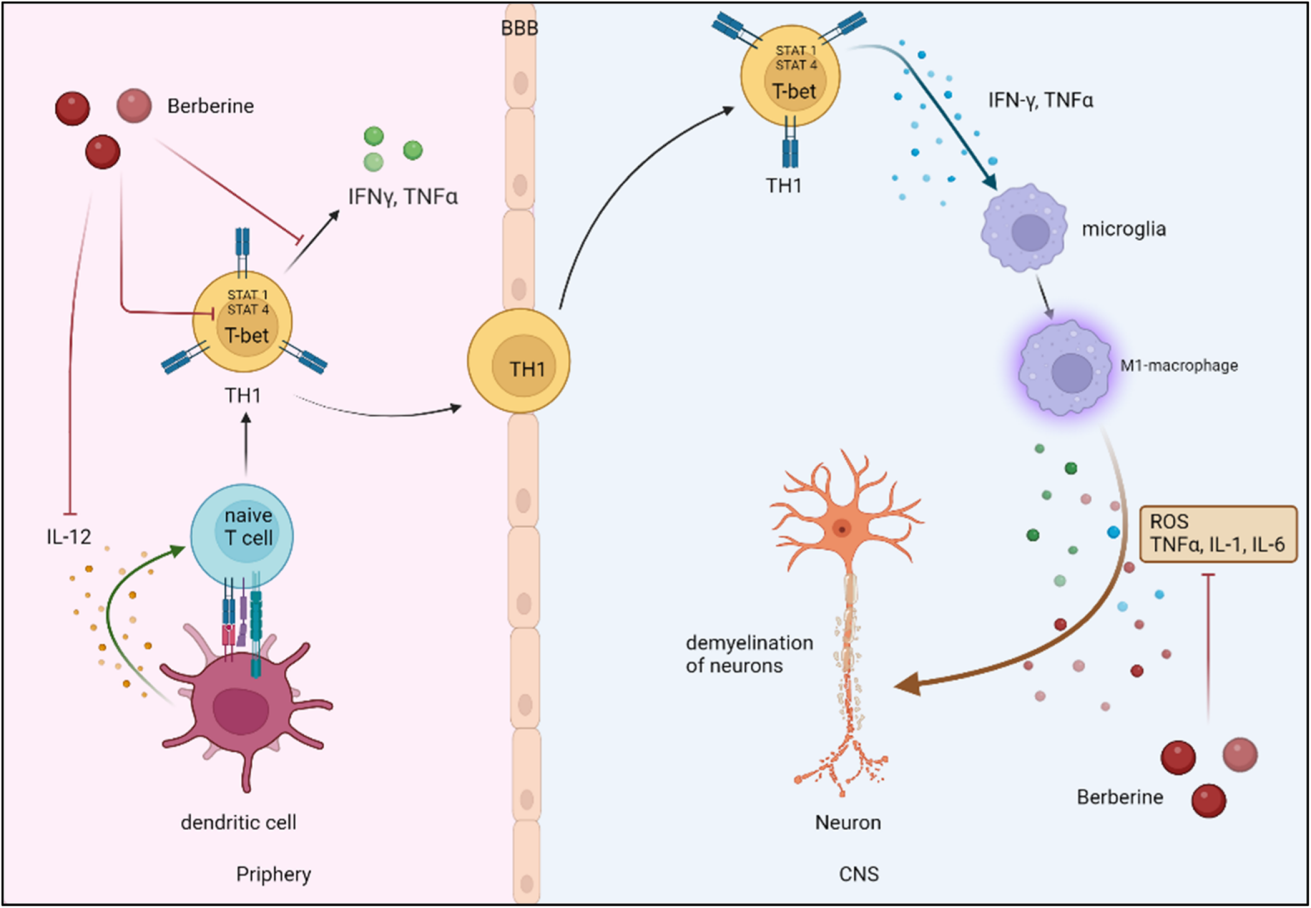
Naïve T cells differentiate into Th1 by presenting the antigen and producing cytokines like IL‐12 from dendritic cells. Th1 cells can cross the blood–brain barrier (BBB) and escape from immune system regulatory mechanisms. As a result, it activates CNS‐resident macrophages (microglia) by generation of cytokines like IFN‐γ and changes them into M1‐phenotype cells that can cause demyelination of neurons by producing ROS and inflammatory cytokines. Berberine can reduce the impact of IL‐12 on naïve T cells, which in turn decreases the expression of STAT 1, STAT 4, and T‐bet and consequently causes the reduction in the Th1 populations. Additionally, berberine can limit the production of inflammatory cytokines from Th1 and M1‐macrophages and also neutralize the effect of ROS produced by M1‐macrophage. CNS, central nervous system; ROS, reactive oxygen species.

Additionally, Th1 is primarily responsible for the development of autoimmune disorders like MS.^56^ The autoreactive Th1 cells can penetrate CNS endothelium and run away from the regulation mechanisms of the immune system and directly or indirectly destroy the oligodendrocytes, myelin sheaths, and axons. Indirect damage is caused by the production of pro‐inflammatory cytokines such as IFN‐γ and IL‐2^57^ (Figure 4).

IFN‐γ, as a signature cytokine produced by Th1 cells, is an important factor in the progression of MS.^58^, ^59^ All CNS cells have IFN‐γ receptors, which means they could be able to react to this cytokine.^60^ Some studies have revealed that MS patients exhibit elevated amounts of TNF‐α and IFN‐γ generated by the Th1 cell type.^61^ Furthermore, other studies showed that MS patients with active lesions had higher IFN‐γ values.^62^ MS risk may rise due to IFN‐γ gene polymorphism.^63^, ^64^ Th1 can induce the activation of CNS resident microglia, and cause the distinction of these cells to inflammatory phenotype (M1‐like) through the production of IFN‐γ.^65^, ^66^ This activated macrophage can participate in the progress of the disease via the generation of inflammatory cytokines and reactive oxygen species (ROS).^67^

Inconsistently displayed that Th1 may act as a protective agent in the pathogenesis of MS. A study done on EAE animal models, observed that animals with IFN‐γ defects suffered from a more severe form of the disease compared to animals with normal amounts of IFN‐γ.^68^, ^69^ Moreover, Ni et al, find out that IFN‐γ leads to the sustainability of the blood–brain barrier (BBB).^70^

Nicotinamide adenine dinucleotide (NAD) levels are influenced by Th1‐generated cytokines such IFN‐γ, IL‐1, and TNF‐α through induced activation of indoleamine 2,3‐dioxygenase (IDO) in the APCs such microglia, macrophage, and DCs during chronic CNS inflammation.^71^ IDO discharges the tryptophan, a substrate for NAD synthesis, and causes the reduction of its extracellular concentration.^71^ A shortage of extracellular tryptophan causes decreasing in the proliferation of autoreactive T cells.^72^, ^73^ However, contradictory abnormal excessive IDO activity might miss neuronal CNS cells famished for external NAD sources. Therefore, demyelination recovery in MS depends on maintaining a homeostatic balance of IDO activity.^71^

Berberine can prevent the differentiation of naïve CD4^+^ T cells into Th1 via decreased phosphorylation of STAT1 and STAT4 that activated following IL‐12 signaling, as well as suppression of T‐bet representation.^23^, ^74^, ^75^, ^76^ It was demonstrated in EAE mice that berberine therapy resulted in a decrease in the number of Th1 cells by blocking the effects of IL‐12 signaling on naive T cells.^75^ The animal model of autoimmune peripheral demyelinating diseases like Guillain–Barre syndrome, known as experimental autoimmune neuritis (EAN), also revealed that berberine can suppress the production of Th1 cytokines such as TNF‐α and result in a reduction in the intensity of the sickness.^77^ Inversely, it was found that berberine may cause Th1 differentiation by inducing the production of IL‐12.^78^ It is thought that these actions of berberine lead to the treatment of disorders like asthma, in which Th2 plays a prominent role.^79^

## 5 ROLE OF TH17 CELLS IN THE PATHOGENESIS OF MS

Th17 cells confer powerful protective immunity to infectious organisms; they also have been found to play crucial pathogenic roles in the inflammatory response and demyelinating lesions within the CNS of MS patients.^80^ Th17 cells are identified by the production of their hallmark cytokine IL‐17A and the expression of the transcription factor ROR‐γt.^81^ In addition to IL‐17, Th17 cells produce other inflammatory cytokines such as IL‐21 and IL‐22.^82^

TGF‐β, in combination with pro‐inflammatory and pleiotropic cytokines such as IL‐6 and IL‐23, is required to induce Th17 cell lineage commitment via expression of transcription factor ROR‐γt.^83^, ^84^ The pathogenic role of Th17 cells in autoimmune diseases has emerged from studies that indicate that IL‐17‐deficient mice are lowly susceptible to EAE, and treatment with IL‐17R antagonist or IL‐17 neutralizing antibody led to the amelioration of EAE.^21^ Additional supporting data for the Th17 came from the study showing that Th17‐axis cell phenotypes and IL‐17 concentration are increased in MS patients with active disease in comparison to healthy subjects.^82^, ^85^ Further evidence demonstrated that augmented expression of IL‐17 was observed in patients in both brain lesions and mononuclear cells isolated from blood and cerebrospinal fluids.^86^ More recently, an increase in the production of IL‐17 is also observed in lymphocytes derived from mice with EAE.^86^, ^87^ Consistent with these observations, IL‐17 transcript is upregulated in MS lesion^62^ and MS activity is correlated with an increased number of Th17 cells in the patient's blood.^88^ The pathogenic effect of Th17 in the EAE model is further confirmed by findings that deficiency or neutralization of IL‐17A delays the onset and reduces the incidence and severity of relapses.^86^

Other proposed pathological functions of IL‐22 and IL‐17, two of the cytokines produced by Th I7 cells, include the disruption of the blood‐brain barrier in vitro and in vivo and the killing of human neurons.^82^, ^89^ These results are in line with our recent data showing effector molecules secreted by Th17 cells induce a strong pro‐inflammatory response and suppress an anti‐inflammatory response in astrocytes^90^ and have been associated with disease onset, progression, and relapse.^91^

Th17 responses are critically involved in the pathogenesis of several autoimmune diseases, so reviewing the effects of Berberine on these cells can be valuable for the treatment of these diseases. An experiment conducted in EAE suggested Berberine can inhibit the differentiation and function of Th17 cells through direct actions on the JAK/STAT signaling pathway; in this way, Berberine downregulates STAT3 phosphorylation and ROR‐γt expression, during Th17 cells differentiation. In addition, Berberine indirectly influences Th17 cells by inhibition of NF‐kB activity in CD11b^+^ APCs, which correlates with the downregulation of costimulatory molecules and suppression of cytokine IL‐6 production; therefore, Berberine can improve EAE.^75^ Another study confirmed that Berberine directly suppressed IL‐17 production by Th17 cells and differentiation of Th17 cells via its indirect effect on DCs from Vogt‐Koyanagi‐Harada patients.^47^ In type 1 diabetes, the Berberine treatment of NOD mice prevented Th17 differentiation by increasing ERK1/2 activity, which implies that ERK may have a negative regulatory role in Th17 differentiation. ERK inhibits Th17 Differentiation by downregulation of STAT3 and ROR‐γt signaling pathway.^92^ Research directed by Mengfan Yue et al. has shown that oral Berberine improves collagen‐induced arthritis (CIA) in rats, an animal model for human rheumatoid arthritis, by suppressing Th17 cell responses, which was correlated with the stimulation of cortistatin (an immunoregulatory neuropeptide) generation from the gut. Intestinal mRNA and protein levels of cortistatin enhanced in Berberine‐treated rats and it negatively attenuated the systemic responses of Th17 cells.^93^ Further evidence also characterized that Berberine could decline excessive responses of Th17 cells and the levels of Th17 cytokines in a rat model of experimental autoimmune myocarditis (EAM), so EAM ameliorated by BRR. The effect of Berberine on Th17 cells is mediated by the downregulation of STAT3 phosphorylation.^74^ Another study on mice with EAE indicated that Berberine suppresses the differentiation of naive CD4+ T cells into pathogenic Th17 cells through direct actions on the JAK/STAT pathway, resulting in the amelioration of inflammation in EAE‐induced mice.^75^ We are confirmed by a study on EAE mice that showed treatment with Berberine significantly downregulates the expression of transcription factors (RORγt) and pro‐inflammatory cytokine derived from Th17 such as IL‐17, which was accompanied by reduced CNS inflammation and demyelination.^23^ Collectively, results from studies point to the promising role Berberine has in suppressing pathogenic Th17 cells in EAE.

## 6 ROLE OF TH2 CELLS IN THE PATHOGENESIS OF MS

Naive T cells can be induced to differentiate Th2, a distinct subset of Th cells characterized by expression of the transcription factor (GATA‐3), which is associated with humoral immunity and allergic immune response.^94^ Effector Th2 cells secrete a profile of potent anti‐inflammatory cytokines, including IL‐4, IL‐5, IL‐10, and IL‐13, and have been implicated in the regulation of autoimmune diseases.^94^ Th2‐associated cytokines tend to mediate anti‐inflammatory humoral response and immune suppression via the inhibition of Th1 cytokine production.^95^ Recent studies have shown that among the T effector subsets not only Treg, but also Th2 cells contribute to recovery from disease by controlling the expansion and activation of autoreactive CD4^+^ T effector cells.^96^ It is noteworthy that the Th1 and Th2 subsets mutually antagonize one another's function and breaking the Th1/Th2 balance with a predominance of Th1 was considered to be important in the initiation and perpetuation of autoimmunity of MS. Additionally, a shift from a Th1 towards a Th2 cytokine profile have been associated with inflammation reduction and improvement of autoimmune conditions.^97^, ^98^

The protective role of Th2 cytokines against inflammatory disease comes from studies that indicate enhancement of a Th2 cytokine will ameliorate or prevent inflammatory disorders, whereas decreased production of Th2 cytokines may be involved in the pathogenesis of several inflammatory disorders.^99^, ^100^ In addition, the adoptive transfer of myelin proteolipid protein (PLP)‐specific Th2 cells has been demonstrated to inhibit ongoing EAE and as well as abrogate established disease.^101^, ^102^ Furthermore, recovery from EAE is associated with an increase in the expression of IL‐4 and Th2 cell production in the CNS, further supporting the hypothesis that Th2 cells can prevent the propagation of inflammation in EAE/MS.^101^, ^102^, ^103^ The mechanisms by which Th2 cells suppress EAE control the development and activity of encephalitogenic Th1 cells and the induction of the alternative (M2) type of macrophages/microglia.^94^, ^104^

Berberine could promote the differentiation of Th2 cells which play a protective role in disease development and alleviating autoreactive inflammatory responses.^21^, ^23^ The potential for Berberine to favor Th2 type response may be an effective therapeutic agent to ameliorate Th1‐skewed inflammatory and autoimmune diseases, such as MS, RA, Crohn's disease, and type 1 diabetes.^105^

An in vitro study demonstrated that Berberine exhibits its immunomodulatory and anti‐inflammatory potential by skewing the Th1/Th2 balance toward the Th2 polarization in mouse primary splenocytes.^105^ This anti‐inflammatory effect is mediated by upregulating the ratio of Th2 (IL‐4)/Th1 (IL‐2) cytokines relative expression.^105^ Investigation of Berberine administration on modulating the immune response in the experimental model of MS showed that the expression of transcription factors and Th2 and Treg cytokines such as IL‐4, STAT6, GATA3, IL‐10 in splenocytes and lymph nodes from EAE mice is significantly increased in Berberine ‐treated groups.^23^ Shifting the balance of Th1/Th2 towards Th2 is associated with reduced CNS inflammation and demyelination in treated groups.^23^ Therefore, Berberine could be helpful in treating autoimmune and inflammatory diseases by shifting the Th1/Th2 balance toward Th2 polarization.

## 7 ROLE OF TREG CELLS IN THE PATHOGENESIS OF MS

Treg cells have anti‐inflammatory effects and maintain tolerance to self‐antigens.^55^ In terms of immunology, MS is associated with the dysfunction of Treg and increases the response of Th1 and Th17 cells.^106^ Research has determined that the regulatory activity of CD4^+^ CD25^hi^ T‐cell is impaired in MS patients.^107^ FoxP3 is a specific transcription factor of Treg cells that plays a crucial role in the development and function of these cells.^108^ The brain biopsy of patients with MS has shown that 30% of the lesions do not express FoxP3. Fas a cell surface receptor for apoptosis, has been shown that increased Tregs in MS brain biopsies indicate enhancement of the susceptibility to apoptosis.^109^ Disruption of Treg cell function during the development of the disease can be modulated by CD28 and CTLA‐4. The research in EAE has identified that expression of CD28, colocalizing with T cell receptor subunit CD3, increases during relapse.^110^ Use of IDO metabolite, which leads to an increase in Treg cell number, leads to considerable improvement of MOG‐induced EAE.^111^

In relapsing‐remitting EAE models, Treg cell depletion can increase the severity of the acute phase of the disease and prevent its recovery.^112^, ^113^ Some MS‐related studies have indicated that although the number of Treg cells is normal, they have functional deficiencies, and in patients with MS, Treg cells have less power to suppress IL‐17 production compared to healthy subjects. Other studies show a two‐ to threefold reduction in the number of Treg cells in the MS exacerbation phase and an increase in the number of these cells in the regression phase, and there is an inverse relationship between the severity and duration of the disease with the number of Treg cells.^114^, ^115^ Regulatory activity of the Treg‐producing IL‐10 cells is characterized by the transfer of IL‐10‐producing Treg cells to EAE mice, and treatment with IL‐10 antagonists also increases the severity of EAE.^116^

The results of a recent study suggest that Treg cells with the expression of the transcription factor Forkhead box protein A1 (FOXA1) play a role in controlling the immune response. FOXA1 is responsible for the expression of Programmed cell death ligand 1 (PD‐L1), which mediates the destruction of T‐cells activated by binding to the PD‐1R receptor; therefore, the active transfer of Treg FOXA1 cells inhibits EAE.^117^ Treg cells protect neurons by direct induction of apoptosis in the inflammatory microglial cells of M1, as well as the change in the phenotype of these cells towards M2 macrophage.^118^

Another study has shown that Treg cells enhance the expression of astrocyte‐derived neurotrophic factors, the brain‐derived neurotrophic factor, and the glial cell‐derived neurotrophic factor, which promotes remyelination and brain repair. It also results in significant improvement in the function of the neural cells with their ability to inhibiting produce of ROS and glutamate.^119^, ^120^

In general, promoting the activity of Treg cells or using their cytokines to treat MS disease is worthy of attention.

Recent research has suggested the use of Berberine as a cure for some autoimmune diseases such as EAE,^121^, ^122^ Type 1 diabetes,^123^ and Rheumatoid arthritis.^124^ The effect of Berberine on spleen tissue Treg cells has been studied by our lab. The results of this study showed that Berberine increases the activity of Treg cells and also induces the production of TGF‐β and IL‐10 by splenocytes.^23^ As reported by Li et al., The differentiation of Th1 and Th17 cells was significantly suppressed by the addition of Berberine, while Berberine did not have a significant effect on Treg cell differentiation.^125^

## 8 ROLE OF OTHER IMMUNE CELLS IN THE PATHOGENESIS OF MS AND MODULATORY EFFECTS OF BERBERINE ON THESE CELLS

In recent years, it has been discovered that there is a distinct subset of IL‐9‐producing effector CD4^+^ T cells. Even though the fact that IL‐9 was formerly connected to a Th2 response, recent research has redefined IL‐9‐producing CD4^+^ T cells as Th9 cells^126^, ^127^ Comparing mice with EAE receiving Th9 cells to mice receiving Th1 and Th17 cells, showed that mice receiving Th9 cells exhibited less lymphocyte infiltration in the meninges.^128^ Additionally, the protective function of IL‐9 was demonstrated in IL‐9R knockout mice, which exhibited a severe form of EAE.^129^ Also, Ruocco et al. discovered an inverse relationship between IL‐9 levels in the CSF of RRMS patients and measures of inflammatory activity, neurodegeneration, and the development of MS‐related disability.^130^ However, some studies showed that IL‐9 neutralization and IL‐9R deficiency reduced EAE^131^, ^132^ It has been observed that adoptively transfer of PLP_180‐199_ peptide‐specific T cells from wild‐type mice or immunization with the PLP_180‐199_ peptide resulted in significantly less severe EAE and lower levels of IL‐17 and IFN‐γ in IL‐9 knockout mice compared to wild‐type mice.^133^ However, the role of Th9 and IL‐9 in the pathogenesis of MS is not clearly defined.

Th22 is characterized by the release of several cytokines, including the most significant one, IL‐22, as well as IL‐13 and TNF‐α.^134^ Beyeen et al showed a connection between the IL‐22Rα2 gene and an increased chance of MS.^135^ Additionally, patients with MS and neuromyelitis Optica (NMO) showed an increase in IL‐22 and Th22 cells.^136^ Kebir et al showed that the IL‐22R was overexpressed in the brains of MS patients. They found that IL‐22 and IL‐17A worked together to damage the integrity of BBB tight junctions by lowering the expression of occludin in endothelial cells.^89^ More recent research revealed that relapsing MS patients had higher serum levels of IL‐22 than healthy donors,^136^, ^137^, ^138^, ^139^ while during the recovery phase of acute EAE, IL‐22 levels decreased.^140^ However, another study showed that mice lacking IL‐22 are completely susceptible to EAE induced by the MOG_35‐55_ peptide.^141^ Therefore, more research is required to determine the exact role of IL‐22 in autoimmune inflammatory disorders of the CNS.

Although much focus on MS pathology has centered on CD4 T cells, data suggest that CD8 T cells play a role in MS. Studies have suggested that the MBP‐specific CD8^+^ T lymphocytes may increase inflammation in the brain.^142^, ^143^ The primary lymphocytes found in the CNS brain lesions of MS patients and mice with EAE were found to be CD8^+^ T cells.^142^ EAE in mice might be induced by the adoptive transfer of CD8^+^ enriched MOG‐specific T cells.^54^ In another investigation, it was discovered that people with MS had more circulating CD8^+^ CD20^+^ T memory cells that were specific to myelin antigens than control subjects. These T cells may be very pathogenic since CD20 expression on them is connected with the upregulation of activation markers, pro‐inflammatory cytokines, and adhesion molecules. Adoptive transfer of CD20^+^ T cells in EAE mice, which led to the degradation of brain tissue integrity and exacerbation of disease severity, supported this concept. Following anti‐CD20 treatment, the fraction of memory and CD20^+^CD8^+^ T cells that are specific for myelin antigens was considerably decreased, showing that the deletion of CD20^+^ T cells has therapeutic promise for MS.^144^ Cytokine‐expressing CD8 T cells are also related to MS. In contrast to inactive lesions, where only a small number of CD8 T cells were IL‐17 positive, active MS lesions had an increase in these cells in the perivascular regions.^145^ All of these CD8 T cells that secrete IL‐17 are members of the CD161^high^ subset of CD8 cells.^146^

NKT cells, also known as natural killer T cells, are a heterogeneous group of T cells that express NK cell surface antigens. Type I NKT, also known as invariant NKT or iNKT, type II (nonclassical) NKT, and NKT‐like cells are the three main populations of NKT cells.^147^ iNKT cells have an invariant Vα24Jα18 T‐cell receptor and identify self‐ and foreign‐derived lipids provided by CD1d as cognate antigens.^148^, ^149^ Changing the iNKT cell population in the peripheral blood of MS patients does not follow a certain pattern in different forms of MS or during different treatments of the disease,^150^, ^151^, ^152^ but there is probably a frequency change among different iNKT cell subsets.^153^ Depending on the form of MS, cytokine production in subtypes of iNKT cells is different.^150^, ^153^, ^154^ iNKT cells may perhaps be a mediator of the immune regulatory impact of IFN‐β therapy in MS.^155^ It was reported in several studies that iNKT cells can protect mice from EAE by modulating CD4^+^ Th1‐ and Th17‐mediated immune responses, but with different mechanisms.^156^, ^157^, ^158^ Besides, some studies show that iNKT cells are more protective against the myelin‐specific Th1 responses after stimulation with alpha‐galactosylceramide (α‐GalCer).^159^, ^160^, ^161^ Also, another research demonstrates that after receiving α‐GalCer, iNKT cells expressing FoxP3 showed protective effects in EAE.^162^

γδ T cells are a discrete group of lymphocytes that express TCR, which is made up of two glycoprotein chains on the surface known as the γ and δ TCR chains. Several studies have reported that γδ T cells present in MS plaques of MS patients (with enrichment to a TCR‐restricted repertoire)^163^ and an increase in γδ T lymphocytes in the CSF, which is associated with their rise in peripheral blood.^164^ γδ T cells isolated from the CNS can be expanded, but only in individuals with the recently developed disease and not in those with chronic MS; whereas expansion of CD16^+^ cytotoxic γδ T cells in MS patients was mainly seen during the progressive phase of the disease.^165^ γδ T cells may play a part in demyelinating diseases; however, this is unclear.

Whether Berberine can affect these cells has not yet been discovered. Hence more investigations need to understand how berberine can affect these cells to change the MS state.

## AUTHOR CONTRIBUTIONS

Esmaeil Yazdanpanah, Sepehr Dadfar, Alireza Shadab, Niloufar Orooji, MohammadHossein Nemati, Alireza Pazoki, and Dariush Haghmorad wrote the main manuscript. Sepehr Dadfar, Niloufar Orooji, and MohammadHossein Nemati designed the figure. Seyed‐Alireza Esmaeili, Rasoul Baharlou, and Dariush Haghmorad reviewed the manuscript and figure.

## CONFLICT OF INTEREST STATEMENT

The authors declare no conflict of interest.
